# Neglect of mental health issues and lack of integration of psychosocial interventions in Zero Leprosy Roadmaps: A critical oversight

**DOI:** 10.1371/journal.pmen.0000140

**Published:** 2024-09-19

**Authors:** Anil Fastenau

**Affiliations:** 1 Marie Adelaide Leprosy Center, Karachi, Pakistan; 2 German Leprosy and Tuberculosis Relief Association (DAHW), Wuerzburg, Germany; 3 Heidelberg Institute of Global Health, University of Heidelberg, Heidelberg, Germany; 4 Department of Global Health, Institute of Public Health and Nursing Research, University of Bremen, Bremen, Germany; 5 Department of Health, Ethics & Society, Faculty of Health, Medicine and Life Sciences, Care and Public Health Research Institute CAPHRI, Maastricht University, Maastricht, The Netherlands; PLOS: Public Library of Science, UNITED KINGDOM OF GREAT BRITAIN AND NORTHERN IRELAND

Leprosy is a poverty-related, infectious neglected tropical disease caused by Mycobacterium Leprae [1]. It is, one of the oldest known diseases with ancient writings dating back to 2000 BC describing those affected by leprosy, which can cause severe irreversible disabilities if not detected and treated early [2]. Global initiatives led by the World Health Organization (WHO) to eliminate leprosy have made remarkable progress, reducing the disease’s prevalence to historically low levels, yet it still occurs in many low- and middle-income countries of the world, despite a steadily declining incidence of new cases globally [3]. The elimination efforts have predominantly focused on the biomedical aspects of leprosy, including early detection, multidrug therapy (MDT), prevention of physical disabilities, and more recently, disease prevention through the use of post-exposure prophylaxis (PEP) with single-dose rifampicin (SDR) [4]. While these are undeniably crucial components of any leprosy control strategy, the neglect of mental health issues and the lack of integration of psychosocial interventions in WHO recommended country owned Zero Leprosy Roadmaps represent a significant gap that must be addressed to achieve truly comprehensive care for persons affected by leprosy.

## The psychosocial burden of leprosy

Leprosy is not just a disease of the body; it profoundly impacts the mental and social well-being of those affected [5]. The societal stigma associated with leprosy is deeply entrenched in many cultures, often leading to social exclusion, discrimination, and significant psychological distress [6]. Persons affected by leprosy frequently experience anxiety, depression, and low self-esteem, which can persist long after the physical symptoms of the disease have been healed [5]. The social stigma can lead to isolation, loss of employment, and strained family relationships, further exacerbating the mental health challenges faced by individuals with lived experience of leprosy [7].

Despite this well-recognized psychosocial burden of leprosy, mental health issues have been largely overlooked in global leprosy control and elimination strategies. According to WHO’s Global Leprosy Strategy 2021–2030 “Towards zero leprosy”, leprosy endemic countries should develop country-owned comprehensive Zero Leprosy Roadmaps to eliminate the disease [8]. However, despite their comprehensive approach, the Zero Leprosy Roadmaps often fall short in effectively addressing the mental health needs of individuals affected by leprosy during implementation. This oversight is particularly concerning, especially considering the increasing recognition of mental health as a critical component of overall well-being and the ongoing efforts to integrate mental health into primary healthcare.

## Integrating mental health into leprosy programs and Zero Leprosy Roadmaps

There are compelling arguments for making mental health and well-being an integral part of leprosy control and elimination efforts. First and foremost, mental health is a critical component of overall health, as recognized by the WHO’s definition of health as a state of complete physical, mental, and social well-being [9]. Neglecting mental health in leprosy control programs and Zero Leprosy Roadmaps undermines the holistic care that individuals with lived experience of Leprosy both need and deserve.

Moreover, untreated mental health issues can hinder the effectiveness of leprosy control programs as they are major obstacles to early detection and treatment adherence [10]. For example, individuals suffering from leprosy-related depression or anxiety are less likely to seek medical care, adhere to multidrug therapy (MDT), attend follow-up appointments, or participate in social reintegration programs [10]. This not only impacts their personal health outcomes but also poses a significant risk to public health efforts to interrupt transmission and eliminate leprosy, as untreated individuals may continue to spread the disease.

## The role of psychosocial interventions

Psychosocial interventions, which may include counselling, peer support, cognitive behavioral therapy, educational interventions, technology supported interventions and community engagement activities, have been shown to be effective in addressing the mental health and social needs of individuals with lived experience of leprosy [11]. These interventions can help reduce stigma, improve mental health outcomes, and facilitate the social reintegration of individuals affected by leprosy [12]. Despite their proven benefits, such interventions are rarely included in national or regional leprosy control and elimination programs.

For instance, in Pakistan, where my research is focused, the Marie Adelaide Leprosy Centre (MALC) has made significant progress in reducing the physical burden of leprosy. However, there is a noticeable gap in the provision of mental health services and psychosocial support for patients. Similar is true for Afghanistan, Bolivia, Tanzania and Togo, where the focus of leprosy control interventions is mainly on the interruption of transmission. This gap is not unique to the aforementioned countries; it reflects a broader global trend where mental health is often sidelined in disease-specific programs.

## Barriers to integration

Several barriers contribute to the neglect of mental health in leprosy control and elimination programs. One of the most significant barriers is the lack of resources and trained personnel [13]. In many countries, mental health services are severely underfunded, and there is a shortage of trained mental health professionals [13]. This makes it challenging to integrate mental health services into existing leprosy programs, which are often already facing challenges of limited resources.

Another barrier is the stigma associated with both leprosy and mental health issues [11]. In many cultures, mental illness is viewed with suspicion and fear [12], similar to the stigma surrounding leprosy. This double stigma can discourage individuals with lived experience of leprosy from seeking help for mental health issues and can also make it difficult to implement mental health programs within leprosy services.

Finally, there is a lack of awareness and understanding of the importance of mental health among policymakers and healthcare providers [14]. This is compounded by a historical focus on the biomedical aspects of leprosy, which has led to the marginalization of mental health in program planning and resource allocation.

## Recommendations for policy and practice

To address these gaps, it is essential to advocate for the integration of mental health services, stigma reduction interventions and psychosocial interventions into leprosy control programs as part of the country owned Zero Leprosy Roadmaps. This can be achieved through several key actions:

1. **Policy Advocacy:** Global health organizations, national governments, and NGOs should prioritize mental health in their leprosy elimination strategies. This includes advocating for the allocation of resources specifically for mental health services within leprosy programs.
2. **Capacity Building:** Training programs should be developed to equip healthcare providers with the skills needed to identify and manage mental health issues in individuals with leprosy. This could involve collaboration with mental health professionals to provide integrated care.
3. **Community Engagement:** Community-based interventions should be expanded to include psychosocial support and mental health education. These programs can help reduce stigma, encourage help-seeking behaviors, and support social reintegration.
4. **Research and Data Collection:** There is an urgent need for more operational research on the mental health impacts of leprosy and the effectiveness of psychosocial interventions. This data is crucial for informing policy and practice and ensuring that mental health is adequately addressed in leprosy programs and Zero Leprosy Roadmaps.
5. **Integration into Primary Healthcare:** Mental health services should be integrated into primary healthcare systems, with a focus on providing holistic care for individuals affected by leprosy. This would ensure that mental health is not treated as an afterthought but as a fundamental integral component of leprosy care.

## Conclusion

The neglect of mental health issues and the lack of psychosocial interventions in Zero Leprosy Roadmaps represent a significant oversight in global efforts to eliminate leprosy. To achieve the WHO’s target of zero leprosy by 2030, it is imperative that mental health be recognized as a critical integral component of leprosy detection and care. By integrating mental health services and psychosocial interventions into leprosy control programs, we can not only improve the quality of life for individuals affected by leprosy but also enhance the effectiveness of efforts to interrupt transmission and achieve a world free of leprosy.
